# Organ sparing of linac‐based targeted marrow irradiation over total body irradiation

**DOI:** 10.1002/acm2.12742

**Published:** 2019-10-11

**Authors:** Gregory R. Warrell, Valdir C. Colussi, Wayne L. Swanson, Paolo F. Caimi, David B. Mansur, Marcos J. G. de Lima, Gisele C. Pereira

**Affiliations:** ^1^ Franciscan Health Cancer Center Munster Munster IN USA; ^2^ Department of Radiation Oncology University Hospitals Cleveland OH USA; ^3^ Department of Medicine–Hematology and Oncology University Hospitals Cleveland OH USA

**Keywords:** equivalent uniform dose, gEUD, TBI, targeted marrow irradiation, TMI, total body irradiation

## Abstract

**Purpose:**

Targeted marrow irradiation (TMI) is an alternative conditioning regimen to total body irradiation (TBI) before bone marrow transplantation in hematologic malignancies. Intensity‐modulation methods of external beam radiation therapy are intended to permit significant organ sparing while maintaining adequate target coverage, improving the therapeutic ratio. This study directly compares the dose distributions to targets and organs at risk from TMI and TBI, both modalities conducted by general‐use medical linacs at our institution.

**Methods:**

TMI treatments were planned for 10 patients using multi‐isocentric feathered volumetric arc therapy (VMAT) plans, delivered by 6 MV photon beams of Elekta Synergy linacs. The computed tomography (CT) datasets used to obtain these plans were also used to generate dose distributions of TBI treatments given in the AP/PA extended‐field method. We compared dose distributions normalized to the same prescription for both plan types. The generalized equivalent uniform dose (gEUD) of Niemierko for organs and target volumes was used to quantify effective whole structure dose and dose savings.

**Results:**

For the clinical target volume (CTV), no significant differences were found in mean dose or gEUD, although the radical dose homogeneity index (minimum dose divided by maximum dose) was 31.7% lower (*P* = 0.002) and the standard deviation of dose was 28.0% greater (*P = *0.027) in the TMI plans than in the TBI plans. For the TMI plans, gEUD to the lungs, brain, kidneys, and liver was significantly lower (*P* < 0.001) by 47.8%, 33.3%, 55.4%, and 51.0%, respectively.

**Conclusion:**

TMI is capable of maintaining CTV coverage as compared to that achieved in TBI, while significantly sparing organs at risk. Improvement on sparing organs at risk permits a higher prescribed dose to the target or the maximum number of times marrow conditioning may be delivered to a patient while maintaining similar typical tissue complication rates.

## 1 INTRODUCTION

Total body irradiation (TBI) for therapeutic purposes had its origin over a century ago. The technique was proposed and referred to during the first decade of the 20th century as an “x‐ray bath” for treatment of malignancies and other diseases with multiple radiation sources.^1^, ^2^, ^3^ It was recognized early on that an extended distance between the radiation source and the patient may decrease superficial dose relative to mean dose within the patient (i.e., the tissue lateral effect), improving the uniformity of the dose distribution and the maximum tolerated dose.^4^, ^5^, ^6^ A further improvement in dose uniformity was achieved with the use of higher energy sources.^5^, ^7^ While oncologic use of TBI up until this point focused on direct treatment of hematopoietic, lymphoid, and other malignancies,^8^ its potential as a conditioning regimen for marrow transplantation was discovered in a series of mid‐century animal experiments, culminating with use of TBI conditioning in leukemia by the Nobel laureate E. Donnall Thomas.^1^, ^9^, ^10^

Dose‐limiting organs in TBI include the brain, eyes, kidneys, liver, and particularly the lungs, which may develop alveolar hemorrhage and radiation pneumonitis.^6^, ^10^, ^11^, ^12^, ^13^ A wide variety of isocentric and extended‐field beam geometries for TBI have been proposed and utilized for providing acceptably uniform dose to the body while sparing the critical organs.^6^, ^11^ By virtue of its nature as an intensity‐modulated radiation therapy (IMRT) technique, helical tomotherapy‐based TBI may be used to spare the lungs and other selected critical organs such as the kidneys and liver;^14^, ^15^, ^16^ some groups have also studied this via linac‐based IMRT.^17^, ^18^ Alternatively, tomotherapy may be take greater advantage of the potential of intensity modulation by providing targeted therapy to the marrow and other hematopoietic and lymphatic organs such as the spleen and lymph node chains, avoiding critical organs outside a defined planning target volume (PTV). This may be done to improve patient outcomes by improving the therapeutic ratio: by intensifying the conditioning dose, decreasing the dose to critical organs, or both.^14^, ^19^, ^20^, ^21^, ^22^

While this technique of targeted marrow irradiation (TMI) may be delivered by helical tomotherapy‐based methods, Aydogan et al. showed that a general‐purpose linac may be used to deliver TMI in a multi‐isocentric technique.^23^, ^24^ This methodology has been adapted at our institution in a Phase I study using TMI in combination with fludarabine and busulfan as conditioning in stem cell transplantation. To find the dose reduction provided by TMI over our institution’s TBI technique and to inform treatment planning for future TMI patients under this protocol, a direct dose comparison was made between dose distributions calculated for both techniques. The dose reduction was quantified by the generalized equivalent uniform dose (gEUD) of Niemierko, used to estimate the equivalent dose to a critical organ or target that, if delivered uniformly to the structure, would lead to the same biological effect as the nonuniform dose distribution delivered.^25^, ^26^

## 2 MATERIALS AND METHODS

A retrospective treatment planning study of TMI vs TBI plans was conducted for ten patients, all but one of them recruited under an institutional review board‐approved phase I clinical trial of TMI preceding administration of fludarabine and busulfan as conditioning for allogeneic stem cell transplantation. Patients were 25–75 yr old at the start of treatment (median of 66 yr), of height 151–183 cm (median of 168 cm), and of weight 50–99 kg (median of 80 kg). The protocol delineates 13 levels of dose escalation for a total prescribed dose from 3 to 18 Gy to determine the maximum tolerated dose. Accordingly, treatment plans with prescribed doses of 3, 4.5, and 6 Gy were generated for three patients, four patients, and three patients, respectively, each with 1.5 Gy per fraction. Up to two fractions were delivered per day (BID), with at least a 6‐h interfraction interval.

The treatment planning and delivery methodology were similar to the multi‐isocentric feathered described by Aydogan et al.^23^, ^24^ Computed tomography simulation images were obtained with the patient in the head‐first supine orientation, scanning from the head to mid‐thigh with a slice thickness of 5 mm. Patient immobilization was done via vacuum bags indexed to the couch and a head and shoulders thermoplastic mask. Three isocenters were marked on the patient: one in the neck, one in the mid‐torso, and one in the pelvis. These isocenters were evenly spaced, with their separation chosen to ensure overlap between the arcs localized to each isocenter. Given the 40 × 40 cm^2^ maximum field size of the linear accelerator for which the treatment plans were generated, and the 30º collimator rotations used, this three‐isocenter technique accommodates a craniocaudal length of 120 cm for the intensity‐modulated portion of the plan. (A 30º collimator rotation was settled upon after experimentation as providing good coverage of the tangential parts of the PTV and acceptable PTV uniformity.)^27^ Due to minimum field overlap required in an isocentric setup, at least one additional isocenter would be used for a craniocaudal length of the intensity‐modulated part of the plan longer than 120 cm. Water‐equivalent bolus (1 cm thick) was placed on the patient’s hands to allow for sufficient dose to the bones of the hands and wrists. An additional set of computed tomography (CT) simulation images was obtained in the feet‐first supine orientation, scanning from the toes to the pelvis.

The plan for each patient was generated for an Elekta Synergy linac equipped with the Agility collimator (Elekta AB, Stockholm, Sweden). The intensity‐modulated part of the plan was generated in Philips Pinnacle^3^ versions 9.10 and 9.14, using a pair of volumetric‐modulated arcs localized to each of the three isocenters (Koninklijke Philips N.V., Amsterdam, Netherlands). The GTV was defined as the spleen and the bone marrow, excluding the mandible to limit complications of the oral cavity. The planning clinical target volume (CTV) was delineated on the head‐to‐thigh CT dataset, consisting of the spleen plus all the bone superior to the mid‐body of the femur, again excluding the mandible. (The bones of the legs and feet were also considered part of the target, but not included in the drawn CTV for the intensity‐modulated part of the plan.) The PTV was defined by a 3 mm expansion from the CTV, trimmed 3 mm from the skin surface but not allowed to clip inside the CTV. The intensity‐modulated part of the plan was generated to cover over 85% of the PTV with 90% of the prescribed dose, limiting the mean dose particularly to the brain, lung, and kidneys each to no more than 50% of the prescribed dose. Dose to other organs outside the PTV, particularly the heart, eyes, oral cavity, and abdominal organs, was limited to as low as practically achievable. The legs were treated by one to two sets of AP/PA parallel‐opposed fields planned on the feet‐first supine dataset, the superior borders of the fields matched to achieve ~1 cm overlap with the inferior edge of the PTV. The field junction between the intensity‐modulated and AP/PA parts of the plan was located within the upper thighs, well away from critical organs. The hotspot at the location of this junction was considered clinically acceptable at the low prescription dose of 3–6 Gy. Both the intensity‐modulated and AP/PA parts of the plan utilized 6 MV photon beams and were calculated on 4 mm dose grids in the Adaptive Convolve dose calculation algorithm.

For each of the isocenters of the intensity‐modulated part of the plan, the patient was lined up according to skin marks made during the simulation procedure, a cone‐beam CT taken, and shifts made to align the visualized skeletal anatomy to that of the simulation CT. If a patient rotation was noted on the localization images, the patient was manually rotated and a new set of localization images taken. The thermoplastic mask was used only for the most superior isocenter, being carefully removed prior to aligning to the other isocenters to make the treatment procedure more tolerable for the patient. After the intensity‐modulated part of the plan had been delivered, the patient was taken off the treatment couch and placed in the feet‐first supine position to deliver the AP/PA plans to the legs.

For each TMI plan, a corresponding TBI plan was also obtained in Pinnacle^3^ for the same CT dataset used to generate the intensity‐modulated part of the plan. This plan consisted of 15 MV AP/PA parallel opposed photon fields with an extended‐field SAD of 530 cm, localized to a point along the axis defined by the TMI isocenters at the level of the umbilicus. As measurements obtained during the commissioning process for the TBI program showed that tissue‐maximum ratios (TMRs) obtained at the extended‐field SAD agreed with those at a SAD of 100 cm to within 2%, no dosimetric corrections were applied to the TBI plans. Furthermore, no attenuating blocks for the lungs or other organs were used in these simulated TBI plans. (An explanation for this choice is in the Discussion section.) Clinical TBI plans at our institution use a 15 MV photon beam to improve homogeneity;^6^ dose uniformity obtained with this AP/PA technique is considered clinically acceptable in our institution without the use of compensators. An in‐house planning worksheet containing the same TMR table of measured tissue maximum ratios is used to calculate the appropriate monitor unit (MU) setting to achieve the prescribed dose in water for the patient’s manually measured separation at the umbilicus. MU settings are calculated in the worksheet as:

$$MU=\frac{Rx\;dose\;per\;fraction/2}{DR_{0}\times TMR_{\mathrm{umb}}\times OAF\times F_{\mathrm{tray}}\times F_{\mathrm{spoiler}}}$$

here, *DR_0_* is the calibrated dose in cGy per monitor unit delivered to the TBI isocenter under reference conditions for TBI (at the center of the field, at the treatment SAD, and at the depth of maximum dose), *TMR_umb_* is the tissue maximum ratio for half the patient separation measured at the location of the umbilicus (the point of calculation in this methodology), *OAF* is the off‐axis factor (1 for the nominal case of the patient's umbilicus along the beam axis), *F_tray_* is the attenuation factor due to the acrylic tray placed at the collimator, and *F_spoiler_* is the attenuation factor of the 1‐cm acrylic spoiler placed in front of the patient to improve beam homogeneity due to scatter as well as a bolus effect.^11^ For the TMI plans, this calculation was used to determine the MU settings that would have been used had these patients been treated with our institution’s standing AP/PA technique; the obtained MU settings were used in the Pinnacle TBI‐simulating plans.

**Figure 1:**
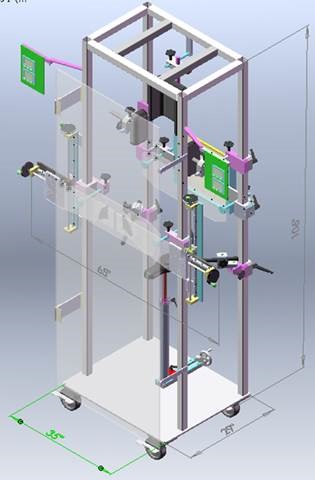
Schematic of the irradiator used for standing AP/PA total body irradiation treatments at our institution (Nogah Engineering LLC, Cleveland, OH). The patient may stand inside the frame behind the acrylic spoiler, facing toward or away from the beam (impingent on the spoiler from the left).

In addition, density overrides within the CT dataset were used to simulate the 1‐cm thick acrylic spoiler used in the standing AP/PA TBI irradiator at our institution (Fig. 1), and to correct for metal artifacts surrounding hip prostheses and dental implants as appropriate. The TBI plans were calculated on a 2 mm dose grid in the collapsed cone convolution (CCC) dose convolution algorithm in Pinnacle, accounting for tissue heterogeneity. To provide an equal comparison between the TMI plans and the TBI plans, both the intensity‐modulated and AP/PA parts of the TMI plan were also recalculated with the CCC algorithm on a 2 mm dose grid. For this comparison, all dose quantities were tabulated and compared as percentages of the prescribed dose, avoiding the effects of different prescriptions on the quantities of interest.

The dose distributions for the TMI and TBI plans were compared in the software package MIM version 6.0. (MIM Software, Inc., Cleveland, OH). DVHs were obtained for each plan type for a variety of structures drawn on the CT images, including CTV, lungs, liver, heart, kidneys, brain, and brainstem. Mean dose, and for some structures, the maximum dose to 0.03 cc were computed for each plan for the studied structures. Additionally, the moderate and radical dose homogeneity indices (mDHI and rDHI) of Oliver et al. were computed for the CTV.^28^ These are defined:

$$\begin{array}{c} {\text{mDHI}\;=\;D}_{95\%}{\text{/ D}}_{5\%}\\ {\text{rDHI}\;=\;D}_{\min}{\text{/ D}}_{\max} \end{array}$$

here, D_95%_ and D_5%_ are the doses to 95% and 5% of the volume, and D_min_ and D_max_ are the minimum and maximum doses, respectively. The studied quantities for the TMI and TBI plans were compared with the paired Student's *t*‐test.

For the CTV, lungs, spinal canal, brain, brainstem, heart, and liver, the generalized equivalent uniform dose (gEUD) of Niemierko was computed, defined:^25^, ^26^

$$gEUD={\left[\sum_{i=1}^{m}\left(v_{i}D_{i}^{a}\right)\right]}^{1/a}$$

here, *v_i_* is the partial volume *i* receiving dose *D_i_*, out of a total of *m* partial volumes, and *a* is the empirical volume parameter (related to the volume‐effect parameter *n* in the Lyman–Kutcher–Burman model by *a* = 1/*n*).^26^, ^29^ Values of *a* from the published literature were used, along with values of *a* varied over a range to assure the robustness of the gEUD comparisons. The gEUD is intended to provide a means of reducing the dose distribution to a structure of interest to an effective dose that, if uniformly delivered to the structure, would result in the same probability of a given biological effect (tumor cure or organ complication) as the nonuniform dose distribution actually delivered. Consequently, values of *a* for the target volumes were negative (resulting in gEUD being dominated by minimum dose to the target), organs at risk considered to have parallel subunits had positive values of *a* close to 1 (producing a gEUD close to the mean dose), and more serial organs at risk had higher values of *a* (giving gEUD closer to the maximum dose).^25^, ^29^

As with the other metrics, gEUDs were compared between the TMI and TBI plans with the paired Student's *t*‐test. Using a similar methodology to that of Xiao et al., a robustness analysis of the differences between gEUDs was done by varying the value of *a* over a wide range.^30^

## 3 RESULTS

**Figure 2:**
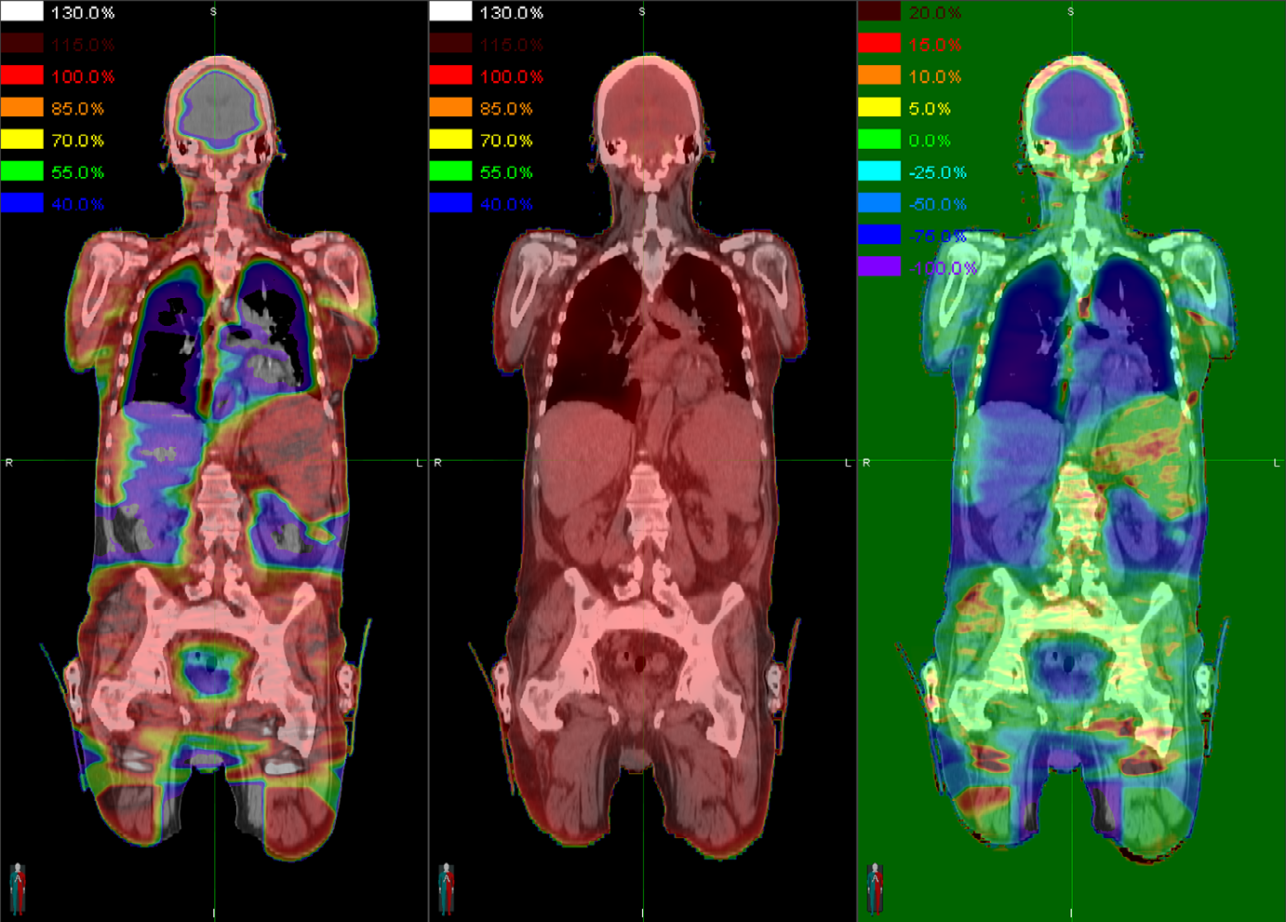
Example comparison of the dose distributions for a targeted marrow irradiation (TMI) plan (left), the corresponding simulated total body irradiation (TBI) plan (middle), and the difference between them normalized to the prescription dose (right), plotted on coronal sections of the computed tomography dataset. For the dose difference image, negative percentages (blue) indicate that the TMI dose is lower than the TBI dose, and positive percentages (red) indicate that the TMI dose is higher. Note the substantial dose sparing of the brain, lungs, liver, kidneys, and other organs in the TMI plan. It is also worth noting that the hotspots occur in different areas between the two plans: there are hotspots in the spleen and in the field junction in the upper thighs for the TMI plan, but in the neck and shoulders for the TBI plan.

**Figure 3:**
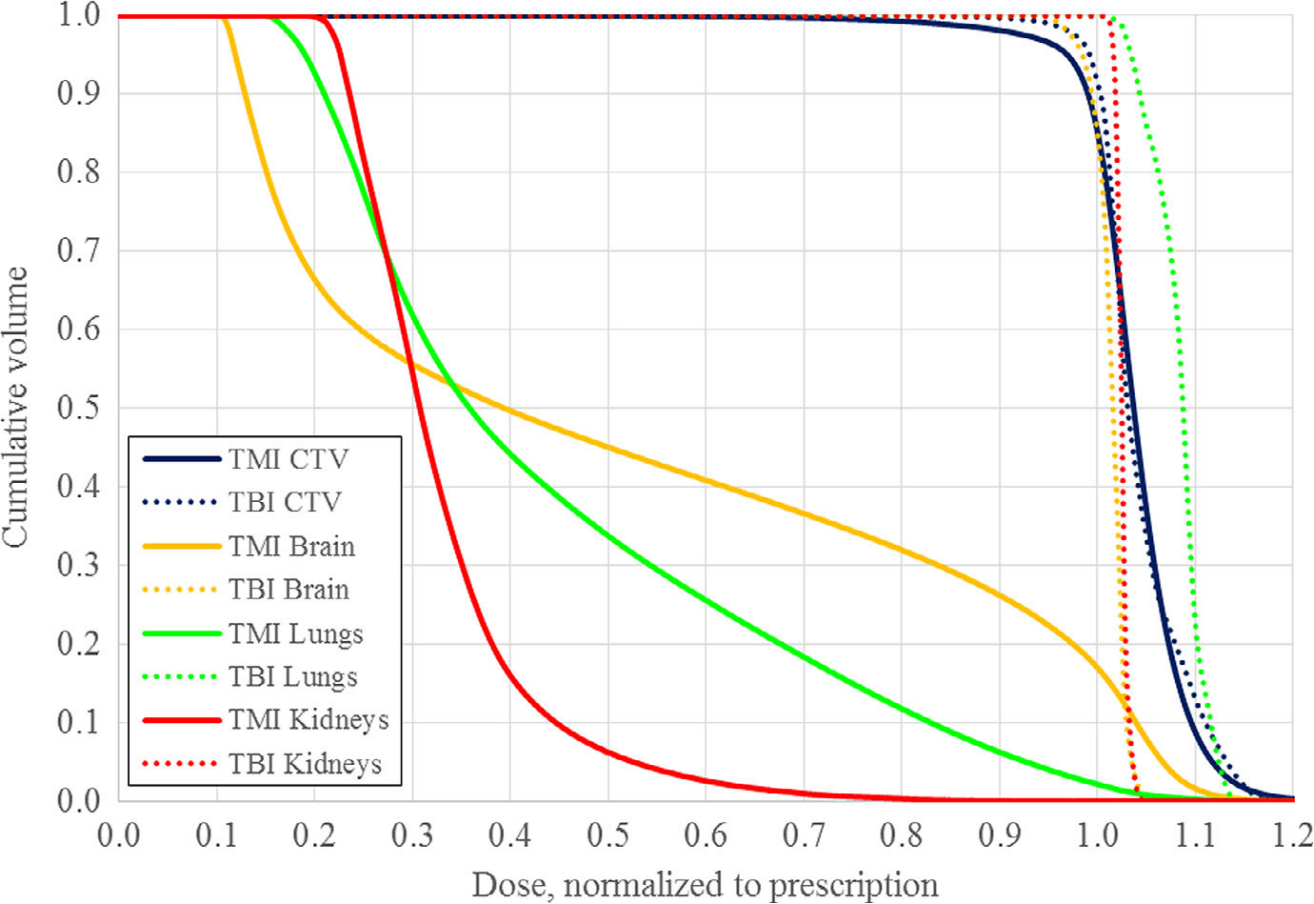
Comparison of the cumulative dose–volume histograms for the clinical target volume (CTV), brain, lungs, and kidneys between the targeted marrow irradiation (solid lines) and total body irradiation (dashed lines) for one of the patients.

The patients in the group had a range of body types, with height ranging from 151 to 183 cm, and body mass index (BMI) ranging from 21.8 to 33.3 kg/m^2^. As expected, the TMI plans provided substantial dose sparing for nearly all studied organs at risk, despite a greater dose heterogeneity than the TBI plans. (See Fig. 2) All TMI plans delivered V_100%_ to over 85% to the PTV and V_50%_ to less than 50% of the brain, lung, and kidneys (See Fig. 3). As anticipated, the TMI plans generally resulted in significantly lower mean dose to the studied organs at risk (OARs) than did the TBI plans, although the maximum dose was typically higher (See Table 1). Between the TMI and TBI plans, no significant differences were found for mean dose, minimum dose to 0.03 cc, D_95%_, D_5%_, or mDHI, to the defined CTV. However, the maximum dose to 0.03 cc and the standard deviation of dose were found to be significantly higher for the TMI plans, and the rDHI significantly lesser. (See Table 2).

**Table 1:** Mean and maximum doses to organs at risks for the targeted marrow irradiation (TMI) and total body irradiation (TBI) plans, as a percentage of the prescribed dose. The percent difference is calculated as the TMI value minus the TBI value divided by the TBI value. *P*‐values that fall below the *P* = 0.05 threshold of significance are bolded for emphasis, and those that fall above this threshold are italicized. Mean doses to breast tissue were calculated for the n = 4 female patients in this study. The mean doses calculated are significantly lower for the TMI plans, except for the spinal canal mean dose, for which there is no significant difference. All maximum doses to organs at risk examined here were significantly greater for the TMI plans.

Quantity	TMI value	TBI value	% difference, *P*‐value
Right lung mean dose	53.3% ± 5.5%	106.6% ± 3.4%	−50.0%, **<0.001**
Left lung mean dose	55.7% ± 5.2%	105.6% ± 3.5%	−47.3%, **<0.001**
Total lung mean dose	54.3% ± 5.3%	106.2% ± 3.5%	−48.8%, **<0.001**
Total kidney mean dose	44.7% ± 5.6%	100.4% ± 3.5%	−55.4%, **<0.001**
Spinal canal mean dose	102.7% ± 2.2%	101.9% ± 3.2%	+0.82%, *0.56*
Spinal canal maximum dose to 0.03 cc	120.0% ± 6.5%	111.2% ± 3.5%	+7.90%, **0.008**
Brain mean dose	66.6% ± 14.1%	99.9% ± 3.8%	−33.3%, **<0.001**
Brain minus PTV maximum dose to 0.03 cc	117.8% ± 6.8%	105.9% ± 4.3%	+11.3%, **<0.001**
Brainstem mean dose	75.8% ± 20.3%	101.2% ± 4.2%	−25.1%, **0.004**
Brainstem maximum dose to 0.03 cc	110.5% ± 4.8%	104.9% ± 4.9%	+5.38%, **0.035**
Heart mean dose	49.3% ± 7.1%	101.3% ± 3.9%	−51.3%, **<0.001**
Heart maximum dose to 0.03 cc	120.5% ± 12.6%	108.7% ± 3.5%	+10.9%, **0.020**
Liver mean dose	49.5% ± 7.5%	99.9% ± 3.9%	−50.4%, **<0.001**
Left parotid mean dose	64.7% ± 18.5%	111.1% ± 4.0%	−41.8%, **<0.001**
Right parotid mean dose	64.9% ± 18.2%	110.9% ± 4.2%	−41.5%, **<0.001**
Total breast mean dose (n = 4)	78.7% ± 6.5%	103.2% ± 4.4%	−23.8%, **0.013**
Skin mean dose	50.1% ± 4.0%	74.7% ± 1.7%	−32.9%, **<0.001**

*P*‐values above the *P*=0.05 threshold of significance are italicized.

**Table 2:** Dose metrics for the clinical target volume (CTV) computed for the targeted marrow irradiation (TMI) and total body irradiation (TBI) plans, as a percentage of the prescribed dose. The percent difference is calculated as the TMI value minus the TBI value divided by the TBI value. *P*‐values that fall below the *P* = 0.05 threshold of significance are bolded for emphasis, and those that fall above this threshold are italicized. For the CTV, the TMI plans demonstrate a significantly higher maximum dose, higher standard deviation of dose, and lower rDHI (indicating greater dose heterogeneity). No significant difference in mean dose or coverage for the CTV was found between the TMI and TBI plans.

Quantity	TMI value	TBI value	% difference, *P*‐value
CTV mean dose	103.8% ± 1.6%	103.0% ± 3.2%	+0.78%, *0.57*
CTV minimum dose to 0.03 cc	35.3% ± 10.1%	42.0% ± 5.8%	−16.0%, *0.076*
CTV maximum dose to 0.03 cc	149.1% ± 13.9%	118.4% ± 4.0%	+25.9%, **<0.001**
CTV D95%	94.8% ± 3.1%	96.8% ± 3.3%	−2.00%, *0.25*
CTV D5%	112.4% ± 2.1%	112.1% ± 3.7%	+0.28%, *0.85*
CTV rDHI (minimum/maximum dose)	0.242 ± 0.080	0.354 ± 0.042	−31.7%, **0.002**
CTV mDHI (D95%/D5%)	0.844 ± 0.028	0.863 ± 0.013	−2.26%, *0.079*
CTV standard deviation of dose	6.12% ± 1.36%	4.78% ± 0.55%	+28.0%, **0.027**

*P*‐values above the *P*=0.05 threshold of significance are italicized.

In all cases, percent differences in quantities between the TMI plans and TBI plans were computed:

$$Difference\;\left(expressed\;as\;percentage\right)=\left(Value_{\mathrm{TMI}}-Value_{\mathrm{TBI}}\right)/Value_{\mathrm{TBI}}$$

Hence, a negative percent difference indicates that the average value is lower for the TMI plans than for the TBI plans.

Table 3 contains gEUDs for the CTV and several studied OARs, given published values of the volume parameter *a*. No significant differences in gEUD were seen either with the CTV or its two parts considered separately, the bones and the spleen. However, a significant improvement in gEUD was observed for every studied OAR, excepting the spinal canal, for which no significant difference was found.

**Table 3:** Calculated values of gEUD for the clinical target volume (CTV) and various organs at risks (OARs), as a percentage of the prescribed dose. The values of the volume parameter *a* and their references are listed. The percent difference is calculated as the targeted marrow irradiation (TMI) value minus the total body irradiation (TBI) value divided by the TBI value. *P*‐values that fall below the *P* = 0.05 threshold of significance are bolded for emphasis, and those that fall above this threshold are italicized. No significant difference in CTV gEUD was found between the TMI and TBI plans. With the exception of the spinal canal, all investigated OARs demonstrated a significantly lower gEUD for the TMI plans.

Structure	*a*, [reference]	TMI value	TBI value	% difference, *P*‐value
Total CTV	−10 31	72.7% ± 22.1%	62.0% ± 27.1%	+17.3%, *0.36*
Bones (part of CTV)	−10 31	72.2% ± 22.0%	61.6% ± 27.3%	+17.1%, *0.37*
Spleen (part of CTV)	−10 31	100.8% ± 4.7%	100.4% ± 3.6%	+0.47%, *0.78*
Total lung	1.2 32	55.4% ± 5.3%	106.2% ± 3.4%	−47.8%, **<0.001**
Total kidney	1.3 32	44.7% ± 5.6%	100.4% ± 3.5%	−54.8%, **<0.001**
Spinal canal	20 32	105.2% ± 2.1%	103.4% ± 3.1%	+1.81%, *0.22*
Brain	4.6 32	83.8% ± 7.5%	99.9% ± 3.8%	−16.1%, **<0.001**
Brainstem	16 32	94.7% ± 7.8%	101.4% ± 4.3%	−6.58%, **0.040**
Heart	3.1 32	57.1% ± 7.5%	101.3% ± 3.9%	−43.7%, **<0.001**
Liver	0.9 32, 33	49.0% ± 7.5%	99.9% ± 3.9%	−51.0%, **<0.001**

*P*‐values above the *P*=0.05 threshold of significance are italicized.

**Figure 4:**
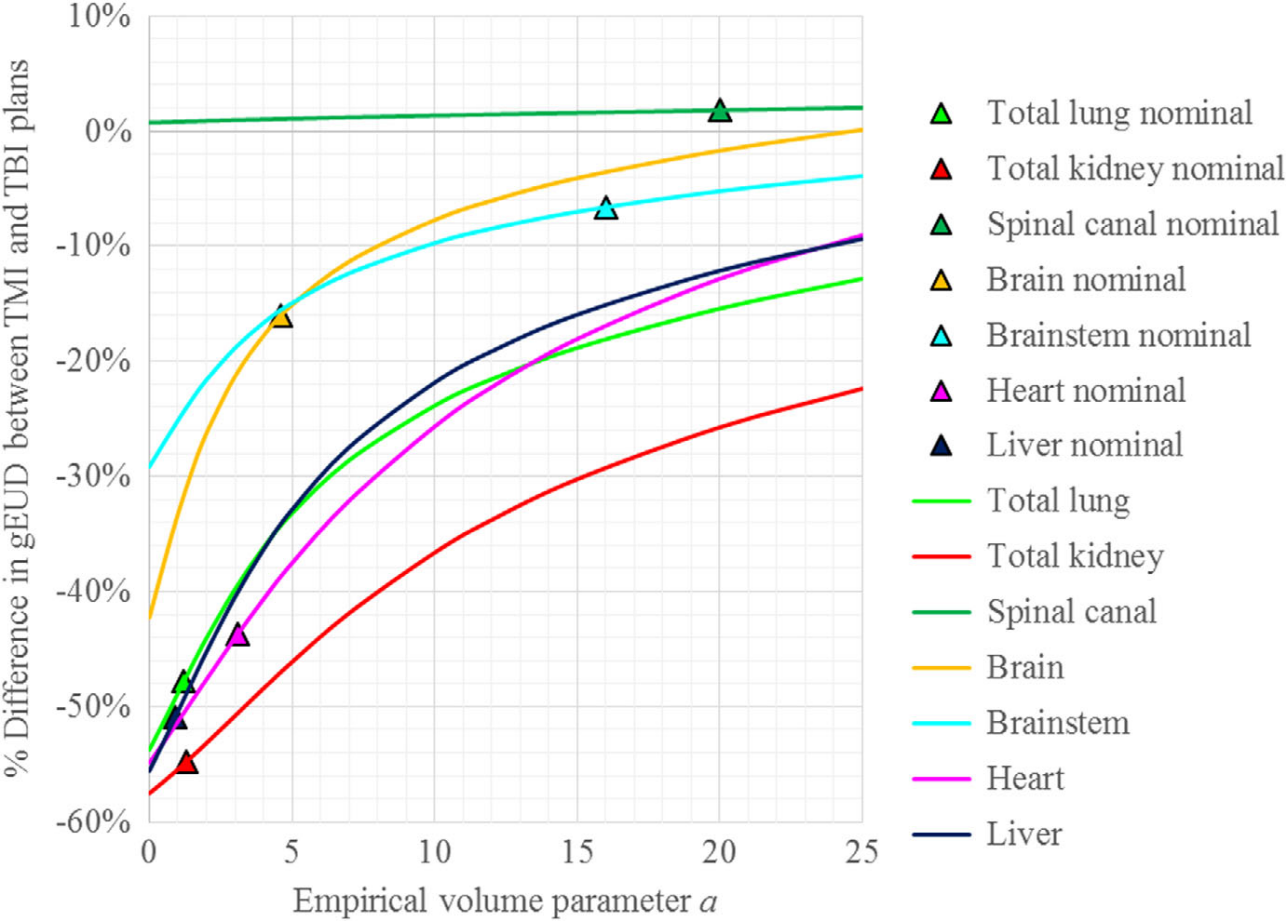
Percent difference in calculated gEUD between the targeted marrow irradiation (TMI) and total body irradiation (TBI) plans, for varied values of the volume parameter *a*. Colored triangles denote the percent difference in gEUD for the nominal (obtained from references) values of *a* used in Table 3. The percent difference is calculated as the TMI value minus the TBI value divided by the TBI value. As stated in Table 5, the differences were all statistically significant (*P* < 0.05) for all organs studied except for the brain for *a* up to 14.7, the brainstem for *a* up to 17.3, and the spinal canal for all studied values of *a.*

Tables 4 and 5 and Fig. 4 present the results of the sensitivity analysis of varied values of the volume parameter *a* on the conclusions of the study. Table 4 demonstrates that even with *a* varied over the range of values typical for target structures, no significant difference could be found between gEUDs calculated for the TMI and TBI plans. Table 5 tabulates the results of a similar analysis for the OARs for whom gEUD was calculated, gauging the robustness of gEUDs with respect to the values of *a* chosen. Figure 4 visually plots the data in Table 5, along with the percent differences in gEUDs between TMI and TBI plans for the nominal values of *a* used in Table 3.

**Table 4:** Calculated percent differences in gEUD for the clinical target volume (CTV) between the targeted marrow irradiation (TMI) and total body irradiation (TBI) plans, for varied values of the volume parameter *a*. The percent difference is calculated as the TMI value minus the TBI value divided by the TBI value. Over the range of values of *a* investigated, no *P*‐values fall below the *P* = 0.05 threshold of significance (non‐significant *P*‐values italicized).

Structure	% difference between TMI and TBI gEUD, *P*‐value			
	*a* = −20	*a* = −15	*a* = −10	*a* = −5
Total CTV	+11.8%, *P = 0.61*	+12.2%, *P = 0.58*	+17.3%, *P = 0.36*	−0.50%, *P = 0.88*
Bones (part of CTV)	+11.8%, *P = 0.61*	+12.1%, *P = 0.58*	+17.1%, *P = 0.37*	−0.66%, *P = 0.84*
Spleen (part of CTV)	−5.03%, *P = 0.11*	−2.01%, *P = 0.41*	+0.47%, *P = 0.78*	+2.11%, *P = 0.16*

*P*‐values above the *P*=0.05 threshold of significance are italicized.

**Table 5:** Calculated percent differences in gEUD for the organs at risks (OARs) between the targeted marrow irradiation (TMI) and total body irradiation (TBI) plans, for varied values of the volume parameter *a*. The percent difference is calculated as the TMI value minus the TBI value divided by the TBI value. *P*‐values that fall below the *P* = 0.05 threshold of significance are bolded for emphasis, and those that fall above this threshold are italicized. The rightmost column reports the value of *a* used in the preceding gEUD study, and the maximum value of *a* still yielding the result of a lower gEUD for the TMI plans with a *P*‐value below 0.05. For most of the OARs studied, the TMI plans had significantly lower gEUDs across a wide range of values of *a.*

Structure	% difference between TMI and TBI gEUD, *P*‐value							Nominal *a*, maximum *a* for *P* < 0.05
	*a* = 0	*a* = 1	*a* = 2	*a* = 5	*a* = 10	*a* = 15	*a* = 25	
Total lung	−53.7%, ***P* < 0.001**	−48.8%, ***P* < 0.001**	−44.0%, ***P* < 0.001**	−33.2%, ***P* < 0.001**	−23.9%, ***P* < 0.001**	−18.8%, ***P* < 0.001**	−12.8%, ***P* < 0.001**	1.2, 50.1
Total kidney	−57.5%, ***P* < 0.001**	−55.4%, ***P* < 0.001**	−53.2%, ***P* < 0.001**	−46.1%, ***P* < 0.001**	−36.6%, ***P* < 0.001**	−30.2%, ***P* < 0.001**	−22.4%, ***P* < 0.001**	1.3, 141.4
Spinal canal	+0.74%, *P = 0.60*	+0.82%, *P = 0.57*	+0.89%, *P = 0.53*	+1.08%, *P = 0.44*	+1.35%, *P = 0.34*	+1.59%, *P = 0.27*	+2.04%, *P = 0.19*	20.0, N/A
Brain	−42.2%, ***P* < 0.001**	−33.3%, ***P* < 0.001**	−26.3%, ***P* < 0.001**	−15.1%, ***P* < 0.001**	−7.72%, ***P = *0.005**	−4.07%, *P = 0.058*	+0.12%, *P = 0.94*	4.6, 14.7
Brainstem	−29.1%, ***P* = 0.004**	−25.1%, ***P* = 0.004**	−21.6%, ***P* = 0.004**	−14.9%, ***P* = 0.005**	−9.73%, ***P* = 0.013**	−6.99%, ***P* = 0.034**	−3.89%, *P = 0.15*	16.0, 17.3
Heart	−54.9%, ***P* < 0.001**	−51.3%, ***P* < 0.001**	−47.6%, ***P* < 0.001**	−37.5%, ***P* < 0.001**	−25.7%, ***P* < 0.001**	−18.1%, ***P* < 0.001**	−9.04%, ***P* = 0.036**	3.1, 26.2
Liver	−55.6%, ***P* < 0.001**	−50.4%, ***P* < 0.001**	−45.2%, ***P* < 0.001**	−32.9%, ***P* < 0.001**	−21.9%, ***P* < 0.001**	−15.9%, ***P* < 0.001**	−9.37%, ***P* = 0.005**	0.9, 34.6

*P*‐values above the *P*=0.05 threshold of significance are italicized.

## 4 DISCUSSION

While other published dosimetric studies have examined TMI delivered via tomotherapy to TBI,^14^, ^34^ the study here presented is the first to directly compare dose distributions for extended‐field AP/PA TBI to those from TMI delivered by a general‐purpose medical linac. The study was partly motivated by the clinical observation that TMI plans, while offering dramatically decreased mean dose as compared to TBI, have a more heterogeneous dose distribution. As pointed out in Table 2, among the ten TMI plans and corresponding TBI plans, the radical dose homogeneity index (rDHI) to the CTV is 31.7% less for the TMI plans (*P* = 0.002), indicating greater dose heterogeneity to the CTV in the TMI plans. Moreover, the standard deviation of dose to the CTV is 28.0% greater in the TMI plans (*P* = 0.027). No significant differences were found in the CTV minimum dose, D95%, or gEUD for a variety of values of *a*, although differences in minimum dose and mDHI to the CTV are close to the *P* = 0.05 threshold of significance. It is possible that significant differences in these metrics would have been uncovered for a larger dataset, resulting in a similar conclusion to that reached for rDHI. Despite these observations, calculation of the gEUD and a corresponding robustness analysis concludes no significant difference in gEUD to the defined CTV. This is likely because within a very large region of interest such as the CTVs studied here (3.9–7.8 L volume of the CTVs), a small volume receiving a dose much higher or lower than the mean is not likely to have a great impact on the gEUD. Small regions of the CTV receiving a particularly low dose in the TMI plans were observed to occur mainly in the bony tissue in and near the nasal passages.

It is highly probable that a more detailed analysis of the dose heterogeneity within the CTV and attempting to calculate the tumor control probability (TCP) would be an oversimplification of the biology of the situation, as the assumption in standard TCP models that target cells are uniformly distributed within a GTV or CTV breaks down in the context of marrow irradiation. It is well established, for example, that the concentration of proliferating hematopoietic cells differs very dramatically between red and yellow bone marrow, yet both marrow types, essentially indistinguishable in normal CT radiography but occupying different locations within the skeletal system, are effectively considered targets of equal importance within the CTV. Various calculation methods of the amount of red bone marrow in a given bone may vary considerably, by a factor of up to 4 for one group.^32^ Moreover, even within red marrow, the cellularity (defined as the fractional volume of hematopoietic cells within the marrow) varies greatly, by a factor of ~3 from one bone to another.^35^, ^36^ Further complications for the patient cohort of this study include their previous histories of radiation therapy and marrow transplantations, as well as concurrently delivered intensive chemotherapy regimens. Nevertheless, studies have shown that the majority of red bone marrow is typically in the spinal column and pelvis, with most of the remainder in the ribs, sternum, cranium, upper limb girdle, and femoral and humeral heads; only a small percentage is in the mandible and distal parts of the limbs.^35^, ^37^, ^38^ Particularly in the vertebrae and pelvic bones, again containing most of the total bone marrow, the dose distributions were visually observed to have acceptable coverage for both the TMI and TBI plans, providing confidence of reasonable dosimetric parity to the marrow between the two modalities. However, it would be impractical or impossible to obtain accurate marrow doses between TMI and TBI without patient‐specific maps of the distribution of active marrow, for example, via SPECT/CT or dual‐energy CT marrow imaging.^39^, ^40^, ^41^

Use of the skeletal bone as the CTV for the marrow target and a 3 mm margin to generate the PTV is typical practice for TMI treatments: as shown in the literature review in Table S1, margins may vary from 0 to 10 mm. This expansion is in addition to the intrinsic expansion in using the bone as the CTV, rather than contouring the marrow within. It is also noteworthy that the defined target and the dose normalization vary considerably among groups. These differences make it challenging to make a direct comparison of target coverage and organ sparing among groups. Nevertheless, as shown in the table, the dosimetric parameters achieved are comparable to those reported by other groups.

The data in Table S1 also show that it is difficult to conclude as to whether tomotherapy‐based or linac‐based TMI provides superior organ sparing, although one group found tomotherapy to provide better sparing based on a phantom study.^27^ Conversely, Aydogan et al. noted superior sparing with their linac‐based technique when the target was revised to correspond to that of a previous tomotherapy dosimetric study.^24^ However, linac‐based TMI has the advantage of availability to institutions that have a standard linac but lack a tomotherapy treatment unit.^17^

Although multiple groups have reported posttransplant relapse as a major detriment to survival in a patient with hematologic malignancies, escalation of the conditioning regimen, either through more intense chemotherapy or an increased TBI dose, has typically increased serious or fatal treatment‐related complications.^42^, ^43^ Targeted marrow irradiation as a component in the conditioning regimen is therefore attractive as a means of escalating dose to hematologic tissues while limiting dose to critical structures to an acceptable level, hopefully improving survival.^14^, ^19^, ^20^, ^21^, ^22^ Nevertheless, there are certain concerns as to the efficacy of radiation delivered via TMI as compared to TBI. The possibility exists that organ sparing accomplished in TMI may also spare clonogens within those organs; clonogens within the circulating blood may also exit the relatively small fields used in intensity‐modulation techniques and “escape” some dose they would otherwise receive.^19^, ^44^ However, Schultheiss et al. point out that dose to spared organs in TMI is not zero, and the density of clonogens is greatly reduced outside the target structures; as the isoeffective dose depends on the logarithm of cell density, this reduced dose may be adequate in the spared organs.^44^, ^45^ As for the “moving target” problem of blood circulation out of the radiation field, a numerical analysis of dose to circulating blood in a model of a serially treated TBI patient suggests that dose heterogeneity to circulating blood elements is minimal for treatment times of 20 min or greater.^46^ We note that while the dimensions and motions of our VMAT fields differ from the modeled tomotherapy field in that study, our TBI treatments take approximately 1 h to deliver per fraction, and also include large AP/PA fields delivered to the legs, likely resulting in highly uniform dose to the blood. Of course, proof of the efficacy of TMI treatments despite these dosimetric concerns must come from clinical studies. Published Phase I studies of TMI and TMLI to date do not suggest the incidence of relapse due to target undercoverage, and a Phase II trial of TMLI is currently underway.^19^, ^43^, ^47^

Based on clinical experience, our institution's application of TBI uses Cerrobend blocks when necessary to limit the lung mean dose to 8 Gy. This dose limit agrees with the clinical heterogeneity‐corrected complication data of Van Dyk et al., which gives a NTCP of ~5% at 8 Gy for radiation pneumonitis following single‐fraction TBI and half‐body irradiation treatments.^13^ At the prescribed TMI dose levels studied in this protocol, 3–6 Gy, lung blocks would not have been used had the patients been treated with TBI instead, so no such blocks were used in the simulated TBI plans. While an additional comparison could have been made between the TMI plans and simulated TBI plans with blocks, this may not have been a completely fair comparison, because at prescription dose levels where blocks would be considered for TBI plans, it is possible that the TMI plans would have been optimized for further lung avoidance than they actually had. In other words, the relative dose distributions in TBI plans might not be independent of the prescribed dose, but might be planned for greater lung avoidance if the prescribed dose is 8 Gy or higher. In summary, it would seem to make the most sense either to compare lower dose TMI plans to unblocked TBI plans (as was done here) or to compare higher dose TMI plans to lung‐blocked TBI plans (an item for future study).

Our institution's TBI methodology allows for opposed‐pair extended‐field delivery (530 cm SAD) in both the standing AP‐PA method discussed above and the supine bilateral (fetal position) method.^6^, ^48^ The former is used less frequently in our clinic due to the increased stress to the patient of having to stand upright for ~1 h but was studied here to take advantage of the nearly whole‐body CT scans obtained for the TBI patients. (In the bilateral method, the patient's arms are placed to shadow the lungs, and the hips are typically flexed so that the head and feet are well within the radiation field; neither of these characteristics is the case for the images used in this study.) For the bilateral method, stacked lead sheet compensators are placed on the collimator block tray to correct for missing tissue of the head and neck and the legs, bringing the dose distributions to within ±10%. In the AP–PA method, compensators are not used owing to the more uniform patient separation in this geometry. For both methods, a 1‐cm thick acrylic spoiler placed adjacent to the patient improves the dose uniformity via scattering and ameliorates the surface dose buildup effect, reducing the 15 MV depth of maximum dose *d_max_* from 2.9 cm to <1 cm. (Fig. 1) Lung blocks, placed close to the patient upstream of the beam, are used to limit lung dose to 8 Gy; further blocking is not used to avoid undercoverage of the ribs. To ensure block positioning, a 35.4 × 43.0 cm^2^ IP type PC lead‐attenuated phosphor portal imaging cassette is placed downstream of the patient for the first 20–25 monitor units of beam delivery (corresponding to ~1 cGy), read off in a FCR Carbon cassette reader (Fujifilm Holdings Corp., Tokyo, Japan), and reviewed before proceeding. To confirm the patient is receiving close to the intended entrance dose, ISORAD n‐type patient surface diode detectors (Sun Nuclear Corporation, Melbourne, FL) are placed at several locations on the patient such as head, thorax, umbilicus, knees, and feet.

The TMI plans provided substantial dose savings to OARs while maintaining acceptable coverage to the target volumes, but tended to have hotspots in excess of those encountered in TBI. Concern over the effect of higher maximum brain dose with the TMI plans (Fig. 3) prompted a gEUD robustness analysis of the brain and several other organs in analogy with that performed for the CTVs. This analysis shows that the gEUD to the brain is significantly less with TMI plans than with TBI plans, for a wide range of values of *a*. It should be noted that the published values of *a* were obtained for treatments with higher prescribed doses and with differing dose distributions than those examined here, possibly limiting their applicability to TMI and TBI treatments. However, the robust improvement in calculated gEUD to the OARs examined over a wide range of examined values of *a* provides confidence that whatever the actual values of *a* are in the context of TMI and TBI, the TMI treatments deliver lower equivalent organ dose and therefore would be expected to lead to fewer complications. For example, gEUD to the total lung is significantly lower for the TMI treatments for *a* ranging from 0 to 50; it does not seem reasonable that the actual value of *a* in the lung for TMI treatments could be outside this extensive range.

As shown in Table 5 and Fig. 4, the percent differences between gEUDs calculated for the TMI plans and TBI plans increase as the value of the volume parameter *a* increases (i.e., the effective dose savings of TMI decreases for high values of *a*). This is explained by the greater hotspots in the TMI plans: as *a* increases, gEUD moves closer to the maximum dose in the structure. A high value of *a* (i.e., more sensitive to hotspots) is typically used for “serial” organs such as the spinal cord and brainstem, while a value close to unity is used for organs of parallel function.^25^ For the nominal values of *a* obtained from the literature, TMI offers a significant dosimetric improvement as predicted by gEUD for all organs investigated except for the spinal canal.

## 5 CONCLUSION

Our institution’s experience with TMI confirms that it is a practical means of delivering targeted pretransplantation conditioning radiation. This direct dose comparison finds no significant difference in coverage, minimum dose, or gEUD to the defined CTV between TMI and TBI treatments, with significant dose savings to nearly all OARs investigated.

## CONFLICT OF INTEREST

No conflict of interest.

## Supporting information


**Table S1.** Overview of prescribed doses, targets, normalization, and achieved dose metrics (where available) for TMI and TMLI plans made available in the literature. The publications are arranged by date of publication, beginning with the most recent. Values with an asterisk were taken from a figure in the publication, rather than from a numerical value in the text or in a table. Values in parentheses indicate a range among cases or tested conditions.
