# Supplementary material for: Organ sparing of linac‐based targeted marrow irradiation over total body irradiation
Source: J Appl Clin Med Phys. 2019 Oct 11;20(11):69–79. doi: 10.1002/acm2.12742 (PMC6839384; doi:10.1002/acm2.12742)
Supplement: Supplementary file 1 — Table S1. Overview of prescribed doses, targets, normalization, and achieved dose metrics (where available) for TMI and TMLI plans made available in the literature. The publications are arranged by date of publication, beginning with the most recent. Values with an asterisk were taken from a figure in the publication, rather than from a numerical value in the text or in a table. Values in parentheses indicate a range among cases or tested conditions. [file ACM2-20-69-s001.pdf]

Table S1: Overview of prescribed doses, targets, normalization, and achieved dose metrics (where available) for TMI and TMLI plans made available in the literature. The publications are arranged by date of publication, beginning with the most recent. Values with an asterisk were taken from a figure in the publication, rather than from a numerical value in the text or in a table. Values in parentheses indicate a range among cases or tested conditions.

Publication	Number of cases	Dose and fractionation	Target and Margin	Additional remarks	Normalization	Brain		Heart		Kidney		Liver		Lung	
						D50	Mean dose	D50	Mean dose	D50	Mean dose	D50	Mean dose	D50	Mean dose
Present study	10	3-6 Gy, 2-4 fractions	CTV: bone (excluding mandible), spleen; PTV: 3mm expansion, trimmed 3mm from skin	Linac (6 arcs); 6 MV beam energy; Pinnacle TPS; clinical treatments	90% of Rx to 85% of PTV	67.9% ± 21.7% (35.9%-96.5%)	66.6% ± 14.1% (45.5%-88.1%)	45.5% ± 7.5% (29.6%-58.4%)	49.3% ± 7.1% (35.2%-59.5%)	41.6% ± 6.3% (30.7%-53.9%)	44.7% ± 5.6% (33.0%-54.3%)	43.9% ± 8.8% (25.4%-59.1%)	49.5% ± 7.5% (35.1% -63.4%)	48.0% ± 6.6% (35.8%-56.3%)	54.3% ± 5.3% (44.7%-60.2%)
[49]	6	10 Gy, 5 fractions	CTV: bone marrow from head to upper third of femurs, except for forearms, brain, liver, spleen and testes; PTV: 5mm expansion from CTV for bone, 3-15mm for other target organs	Linac (6 arcs); 6-15 MV beam energy; Eclipse TPS; non-clinical plans; monitor unit rate varied from 40 to 600 MU/min	100% of Rx to 90% of PTV						(66%-68%)				(57%-59%)
[50]	10	12 Gy, 6 fractions	CTV: bone and central nervous system; PTV: manually expanded to include adjacent soft tissues	Linac (10 arcs); 6 MV beam energy; Eclipse TPS; clincal treatments; obese (BMI > 30 kg/m^2) patients	90% of Rx to 100% of PTV				55% ± 7%		63%		65% ± 5%		66% ± 3%
[50]	10	12 Gy, 6 fractions	CTV: bone and central nervous system; PTV: manually expanded to include adjacent soft tissues	Linac (10 arcs); 10 MV beam energy; Eclipse TPS; non-clinical plans; obese (BMI > 30 kg/m^2) patients	90% of Rx to 100% of PTV				48% ± 3%		54%		54% ± 3%		61% ± 3%
[51]	51	12-20 Gy, 8-10 fractions	Bone and bone marrow (excluding mandible), major lymph node chains (excluding mesenteric and Waldeyer Ring lymph nodes), spleen, testes, liver, and brain (12 Gy to brain)	Tomotherapy; clinical treatments; TMLI plans with varying prescribed dose levels to various target structures	Unspecified in publication			42.2% ± 10.3% (28.8%-69.2%)		37.9% ± 9.2% (21.8%-67.5%)				41.5% ± 6.3% (32.0%-55.0%)	
[41]	13	18 Gy, 6 fractions	CTV: entire skeleton; PTV: 5mm expansion from CTV	Tomotherapy; non-clinical plans (6 patients, 7 cadavers)	85% of Rx to entire CTV						38.8% ± 4.4%				47.1% ± 2.1%
[27]	1	12 Gy, 8 fractions	CTV: all bones, excluding mandible, distal parts of extremities; PTV: 5mm expansion from CTV in ribs and femurs, 0mm expansion elsewhere	Tomotherapy; VoLO TPS; phantom study	100% of Rx to 95% of PTV	60.5%*	60.3%	21.0%*	24.5%	26.6%*	28.8%	39.6%*	41.3%	50.1%*	56.4%
[27]	1	12 Gy, 8 fractions	CTV: all bones, excluding mandible, distal parts of extremities; PTV: 5mm expansion from CTV in ribs and femurs, 0mm expansion elsewhere	Linac (8 arcs); Eclipse TPS; phantom study	100% of Rx to 95% of PTV	100.9%*	99.0%	40.2%*	40.8%	58.1%*	60.0%	62.5%*	63.5%	70.0%*	72.0%
[52]	4	14.4 Gy, 8 fractions	Unspecified in publication	Tomotherapy; non-clinical plans	Unspecified in publication				33.7%		28.1%		42.1%		45.9%
[52]	4	14.4 Gy, 8 fractions	Unspecified in publication	Tomotherapy; non-clinical plans	Unspecified in publication				34.8%		27.9%		41.7%		47.2%
[43]	14	3-12 Gy, 1.5 Gy per fraction	CTV: all bones (including mandible, excluding extremities except for the upper half of the femurs); PTV: 3mm expansion	Linac; Eclipse TPS; clinical treatments	99% of Rx to >95% of PTV			62%*		55%*		58%*		68% (58%-80%)	
[22]	12	12-15 Gy, 9-10 fractions	Target: all bones (excluding mandible, maxillary bones), major lymph node chains, testes, spleen, liver	Tomotherapy; clinical treatments; TMLI plans with varying prescribed dose levels to various target structures	100% of Rx to 85% of target			51.1% (43.9%-57.1%)		46.9% (40.2%-51.8%)		Part of target		45.3% (39.6%-49.9%)	
[24]	6	12 Gy	CTV: bone (excluding forearms and hands); PTV: 3mm expansion	Linac (9 arcs); Eclipse TPS; non-clinical plans	100% of Rx to 95% of PTV	62% ± 3% (58%-65%)		46% ± 6% (38%-53%)		45% ± 5% (40%-51%)		49% ± 1% (48%-51%)		60% ± 3% (57%-63%)	
[24]	6	12 Gy	CTV: bone (excluding mandible, maxillary bones, forearms, and hands); PTV: 3mm expansion	Linac (9 arcs); Eclipse TPS; non-clinical plans	100% of Rx to 85% of PTV	48% ± 3%		41% ± 5%		29% ± 6%		39% ± 2%		47% ± 3%	
[53]	6	12 Gy	PTV: 3mm expansion from skeletal bone	Linac (27 static fields); 6 MV beam energy; Eclipse TPS; non-clinical plans; adults	99% of Rx to 95% of PTV		58.8% (48.6%-62.3%)		60.7% (57.4%-65.0%)		46.6% (43.4%-49.8%)		53.8% (46.9%-59.3%)		60.0% (57.2%-62.5%)
[53]	1	12 Gy	PTV: 3mm expansion from skeletal bone	Linac (18 static fields); 6 MV beam energy; Eclipse TPS; non-clinical plan; child	99% of Rx to 95% of PTV		59.8%		50.8%		34.2%		49.10%		54.2%
[53]	2	12 Gy	PTV: 3mm expansion from skeletal bone	Linac (18 static fields); 6 MV beam energy; Eclipse TPS; non-clinical plan; infants	99% of Rx to 95% of PTV		60.3% (60.3%-60.3%)		54.8% (53.8%-55.8%)		36.0% (33.9%-38.1%)		39.6% (34.4%-44.7%)		53.8% (53.7%-53.8%)
[21]	33	12 Gy, 8 fractions	GTV: bone (excluding mandible, maxillary bones), major lymph node chains, spleen	Tomotherapy; Hi-Art TPS; clinical treatments	100% of Rx to 85% of GTV	57% (34%-101%)		55% (45%-68%)		57% (42%-68%)		62% (57%-68%)		48% (41%-57%)	
[20]	22	10-18 Gy, 5-8 fractions	Target: all bones (excluding mandible, maxillary bones, and extremities except for the humeri)	Tomotherapy; Hi-Art TPS; clinical treatments	100% of Rx to 85% of Target	54% (39%-73%)		49% (43%-55%)		59% (56%-64%)		61% (55%-81%)		46% (37%-56%)	
[19]	13	10-16 Gy, 5-8 fractions	GTV: all bones (excluding mandible, maxillary bones), no expansion	Tomotherapy; Hi-Art TPS; clinical treatments	100% of Rx to 85% of GTV	53% (39%-73%)		50% (47%-55%)		60% (56%-64%)		63% (55%-81%)		49% (44%-56%)	
[19]	8	12 Gy, 8 fractions	GTV: all bones (excluding mandible, maxillary bones), major lymph node chains, spleen, no expansion	Tomotherapy; Hi-Art TPS; clinical treatments	100% of Rx to 85% of GTV	43% (34%-52%)		52% (50%-57%)		56% (50%-62%)		77% (65%-93%)		48% (41%-57%)	
[54]	1	12 Gy, 8 fractions	PTV: all bones in phantom, no expansion	Linac (27 static fields); Eclipse TPS; phantom study	99% of Rx to >99% of PTV	54.8%		35.7%	38%	41.4%	43%	37.1%	39%	52.7%	56%
[54]	1	12 Gy, 8 fractions	PTV: all bones in phantom, 3mm expansion	Linac (27 static fields); Eclipse TPS; phantom study	99% of Rx to >99% of PTV	60.6%		36.4%		42.6%		39.0%		57.1%	
[44]	6	10-20 Gy, 5-10 fractions	GTV: bone (excluding mandible); PTV: expansion for ribs manually contoured from inspiration, expiration, and shallow breathing scans, plus 5mm expansion in arms and thighs	Tomotherapy; Hi-Art TPS; clinical treatments	Unspecified in publication	59%		51%		60%		58%		53%	
[44]	1	12 Gy, 6 fractions	GTV: bone (excluding mandible), spleen, lymphatic system, brain, liver; PTV: expansion for ribs manually contoured from inspiration, expiration, and shallow breathing scans, plus 5mm expansion in arms and thighs	non-clinical plan; TMLI plan to adult	Unspecified in publication			41%		58%				43%	
[44]	1	12 Gy, 6 fractions	GTV: bone (excluding mandible), spleen, lymphatic system, brain, liver; PTV: expansion for ribs manually contoured from inspiration, expiration, and shallow breathing scans, plus 5mm expansion in arms and thighs	non-clinical plan; TMLI plan to child	Unspecified in publication			38%		51%				39%	
[55]	1	6 Gy, 3 fractions	CTV: all skeletal bone; PTV: 10mm expansion	Hi-Art TPS; clinical treatment	Unspecified in publication			70.2%		40.0%		69.5%		56.2%	
[23]	1	12 Gy	CTV: bone (excluding extremities except for upper half of femurs)	Linac (9 arcs); Eclipse/Helios TPS; non-clinical plan	100% of Rx to 92% of CTV (estimated)	61%		59%		57%		54%		58%	
[34]	1	12 Gy, 10 fractions	Target: all bones (excluding mandible, maxillary bones, and extremities except for the humeri)	Tomotherapy; Hi-Art TPS; non-clinical plan	100% of Rx to at least 80% of target	33%		52%		47%		50%		36%	
[34]	1	20 Gy	Target: all bones (excluding mandible, maxillary bones, and extremities except for the humeri)	Tomotherapy; Hi-Art TPS; non-clinical plan	100% of Rx to at least 80% of target	40%		32%		44%		44%		34%	
[34]	1	10 Gy, 5 fractions	Target: all bones (excluding mandible, maxillary bones, and extremities except for the humeri)	Tomotherapy; Hi-Art TPS; clinical plan	100% of Rx to at least 80% of target	47%		48%		60%		60%		44%	
[14]	1	13.2 Gy, 8 fractions	Marrow target: all bones, excluding extremities except for the humeri and femoral heads; PTV: 4mm expansion of marrow target	Tomotherapy; Hi-Art TPS; phantom study	100% of Rx to 95% of PTV			33.6%*		39.4%*		33.6%*		63.1%	
